# The estimated incidence of induced abortion in Kenya: a cross-sectional study

**DOI:** 10.1186/s12884-015-0621-1

**Published:** 2015-08-21

**Authors:** Shukri F. Mohamed, Chimaraoke Izugbara, Ann M. Moore, Michael Mutua, Elizabeth W. Kimani-Murage, Abdhalah K. Ziraba, Akinrinola Bankole, Susheela D. Singh, Caroline Egesa

**Affiliations:** Research Division African Population and Health Research Center (APHRC), APHRC Campus, Manga close off Kirawa road, Kitisuru, P.O. Box, 10787-00100, Nairobi Kenya; Research Division, Guttmacher Institute, 125 Maiden Lane, 7th floor, New York, NY 10038 USA

## Abstract

**Background:**

The recently promulgated 2010 constitution of Kenya permits abortion when the life or health of the woman is in danger. Yet broad uncertainty remains about the interpretation of the law. Unsafe abortion remains a leading cause of maternal morbidity and mortality in Kenya. The current study aimed to determine the incidence of induced abortion in Kenya in 2012.

**Methods:**

The incidence of induced abortion in Kenya in 2012 was estimated using the Abortion Incidence Complications Methodology (AICM) along with the Prospective Morbidity Survey (PMS). Data were collected through three surveys, (i) Health Facilities Survey (HFS), (ii) Prospective Morbidity Survey (PMS), and (iii) Health Professionals Survey (HPS). A total of 328 facilities participated in the HFS, 326 participated in the PMS, and 124 key informants participated in the HPS. Abortion numbers, rates, ratios and unintended pregnancy rates were calculated for Kenya as a whole and for five geographical regions.

**Results:**

In 2012, an estimated 464,000 induced abortions occurred in Kenya. This translates into an abortion rate of 48 per 1,000 women aged 15–49, and an abortion ratio of 30 per 100 live births. About 120,000 women received care for complications of induced abortion in health facilities. About half (49 %) of all pregnancies in Kenya were unintended and 41 % of unintended pregnancies ended in an abortion.

**Conclusion:**

This study provides the first nationally-representative estimates of the incidence of induced abortion in Kenya. An urgent need exists for improving facilities’ capacity to provide safe abortion care to the fullest extent of the law. All efforts should be made to address underlying factors to reduce risk of unsafe abortion.

## Background

Every year, 22 million women worldwide have an unsafe abortion. The majority (98 %) of these occur in developing countries [1]. The 2008 worldwide unsafe abortion rate was 14 per 1000 women aged 15–44 while the rate for Sub-Saharan Africa (SSA) was much higher at 31 per 1000 women of reproductive age [1, 2]. Studies conducted in SSA have shown that induced abortions in the region are generally unsafe as the majority of them are illegal [3–7]. In countries where access to safe and legal abortion is limited, many women with unintended pregnancies resort to unsafe abortions [8].

The risks of unsafe abortion run along a continuum [9] ranging from severe morbidity (hemorrhage, sepsis, organ failure) [10–12] to no complications. While abortion is getting safer worldwide, evidence indicates a higher rate of hospitalization due to unsafe abortion complications for the Eastern Africa region (10 per 1000 women aged 15–44 years) than for sub-Saharan Africa overall (7.5 per 1000 women aged 15–44 years). Sub-Saharan Africa has the highest rates of hospitalization due to unsafe abortion worldwide [13].

Maternal mortality, to which unsafe abortion is a major contributor, is unacceptably high in Kenya according to the last Kenya Demographic and Health Survey (2008–2009), with about 488 maternal deaths per 100,000 live births [14] while the World Health Organization (WHO) estimates 413 maternal deaths per 100,000 live births in 2008 [15]. The WHO estimated the Eastern African region where Kenya belongs to have the highest (18 %) proportion of maternal deaths being attributed to unsafe abortion in 2008 [1]. Another study on maternal mortality in urban slums in Nairobi estimated the maternal mortality ratio in the slums to be 706 deaths per 100,000 live births with 31 % of these deaths being attributed to abortion complications [4]. According to a 2002 study which was conducted before the 2010 constitutional reform, about 21,000 women were admitted annually to public hospitals with abortion complications in Kenya. This translated into an annual incidence of abortion-related complications of 3.0 per 1000 women aged 15–49 years [3].

Until recently, the abortion law in Kenya was highly restrictive and only permitted abortion to save the life of the woman. With the 2010 promulgation of the new Constitution, abortion became a subject of much discussion in the country because the new abortion law states that abortion is permitted if the life or health of the woman is in danger [16]. The clause, “to protect the woman’s health,” is interpreted by some to mean that abortions on-demand are now legal under the Kenyan constitution. This ambiguity remains and providers are unsure of whether they would be protected by the Constitution if they were to provide abortions under the health clause. Following the enactment of the new constitution, no new legislation has been made to offer clarification and provide guidance on the operationalization on what the new legislation in the constitutions seeks to achieve. Furthermore, women seeking abortions are often not aware of conditions under which abortion may now be deemed legal in Kenya [17]. Barriers such as social, cultural and religious beliefs that condemn abortion will continue to limit access even if it were legally permitted [17]. Thus, many unsafe abortions and associated complications are likely continuing to occur in the country and yet the extent of the burden is unknown [1].

The earlier and only abortion incidence study in Kenya was a public hospital-based study conducted in 2004. This study estimated the abortion rate to be 45 per 1000 women of reproductive age (15–49 years) [18]. This study used only a Prospective Morbidity Survey (PMS). It also did not attempt to distinguish between induced and spontaneous abortions and only larger public health facilities were included in the sample. In this paper, we present estimates of the national and regional incidence of induced abortion in Kenya in 2012 based on a representative sample of all health facilities that provide post-abortion care (PAC) in both the public and private sector. By quantifying abortion incidence, this study also provides estimates of the unintended pregnancies in Kenya in 2012.

### Data and methods

Estimates of abortion incidence in Kenya were derived using an indirect estimation method; the Abortion Incidence Complications Methodology (AICM) in conjunction with the Prospective Morbidity Survey (PMS) [19] that involves use of original data collected in three surveys. This approach was recently used by Singh et al. in Ethiopia [7] and Levandowski et al. in Malawi [20].

### Sampling of health facilities

The Kenya Essential Package for Health (KEPH) defines six levels of preventive and curative public and private health service provision which are listed on the Health Management Information System’s (HMIS) Master Facility List [21]. Level I represents the lowest level of health care at community level with no physical infrastructure, level II facilities are dispensaries and clinics, level III facilities consist of health centers and maternity homes, level IV facilities consists of district hospitals, level V facilities consists of regional referral hospitals and level VI facilities provide specialty care and it consists of tertiary referral hospitals.

The Master Facility List obtained January 31, 2012 was used to identify the facilities with the potential to provide PAC^1^ services from both the public and private sector. The universe included 2,838 health facilities from levels II-VI. Due to limited resources, we needed to collapse geographic regions with similar geographical and cultural characteristics to conduct the sampling thus the eight provinces in Kenya were combine into five geopolitical regions namely; 1) Nairobi and Central (N&C) ; 2) Coast and North Eastern (C&NE) ; 3) Eastern (E); 4) Nyanza and Western (N&W) and 5) Rift Valley (RV).

A stratified random sampling approach was used to select the health facilities to be surveyed. The health facilities were stratified by region and level or type of facility. All the level V and VI facilities were included because these facilities are most likely to manage and treat high numbers of abortion-related complications in Kenya. Level II-IV facilities’ sampling fractions varied as follows, depending on the region: level IV health facilities were sampled at 18–36 %; level III health facilities at 10–17 %; while level II facilities were represented at 5–19 % of all facilities in that level and geopolitical region.

The total number of facilities selected was 350 (Table 1) (~12 % of all facilities). Of the 350 sampled health facilities, we obtained complete questionnaires from 328 health facilities for the HFS and 326 facilities for the PMS. Public facilities had a response rate of 99 % while the private for-profit and private not-for-profit sector had response rates of 92 % and 80 %, respectively. The study received very high response rates because the Ministry of Health (MOH) encouraged participation as the MOH was one of the study partners and as such, provided approval letters for the study as well as facilitated the study obtaining approval letters from MOH. Of the 6 % of the facilities that did not participate, reasons for non-response included unwillingness by facility officials to participate in the study for unspecified reasons (5 %) and inaccessibility of the facility to interviewers due to political insecurity (1 %). Of the 94 % of the facilities that agreed to participate in the study, less than 3 % (2 facilities) of the records had missing caseload data which was imputed using the mean computed from non-missing data available from facilities that were in the same level and ownership as the facility to be imputed.

**Table 1:** Distribution of health facilities that answered the HFS by region according to facility level and ownership

Level of facility & Ownership	Health facilities that offer PAC in Kenya^1^						Survey Sampling Fractions						Survey response rate (%)
	N&C	C&NE	E	N&W	RV	Total	N&C	C&NE	E	N&W	RV	Total	
Level of Facility													
Level 2	108	334	156	387	426	1,411	19	6	13	5	5	7	95
Level 3	209	150	136	271	258	1,024	15	17	16	10	11	13	93
Level 4	70	63	56	110	86	385	29	32	36	18	23	26	94
Level 5	5	2	3	4	2	16	100	100	100	100	100	100	88
Level 6	1	0	0	0	1	2	100	-	-	-	100	100	100
Ownership													
Public	189	396	262	532	538	1,917	18	11	19	9	7	11	99
Private for profit	124	99	44	113	100	480	23	11	9	5	11	13	92
Private not for profit	80	54	45	127	135	441	20	22	27	12	16	17	80
Total number of facilities	393	549	351	772	773	2,838	20	12	19	9	9	12	94

Note: *N&C* = Nairobi and Central, *C&NE* = Coast and North Eastern, *E* = Eastern, *N&W* = Nyanza and Western, *RV* = Rift Valley

### Data sources

Data for this study were collected from three different sources: (a) a survey of post-abortion care (PAC) providers in a nationally-representative sample of public and private health facilities (the Health Facilities Survey (HFS)); (b) a survey of patients seeking abortion care presenting over a 30-day period in health facilities (the Prospective Morbidity Survey (PMS)); and (c) interviews with a sample of purposively-selected key informants from different regions of Kenya who are knowledgeable about reproductive health issues including abortion and PAC-related issues (the Health Professionals’ Survey (HPS)). The study protocol received full research ethics board approvals from the Kenya Medical Research Institute (KEMRI), Nairobi (NON SSC No. 320) on the 24th of February 2012 and from the Guttmacher Institute, USA (IRB00002197) on the 18th of April 2012.

### Health Facilities Survey (HFS) and Prospective Morbidity Survey (PMS)

The HFS interviews were conducted with a health professional knowledgeable about post-abortion care at each selected facility. At large facilities, such as referral hospitals, the respondent was the chief of the obstetrics and gynecology department or an obstetrician-gynecologist. At lower level facilities, a nurse, midwife or another health worker in a position to provide information about abortion care in that facility was interviewed. Each respondent was interviewed using a face-to-face structured questionnaire.

Retrospective estimates of the number of in- and out-patient PAC cases treated at that facility in the past month and typical month were obtained from HFS respondents. In the event that the respondent was unable to give the monthly estimates, retrospective estimate of the past year and typical year were requested. HMIS data on the PAC cases treated in that facility were also collected. Other information captured in the survey included the health facilities’ PAC services including infrastructure in place, equipment’s used, and information on family planning counseling services offered to post-abortion care patients visiting the facilities.

The PMS captured data on each woman who obtained abortion-related care in the same health facilities in which the HFS was being conducted during a 30 day period so as to capture abortion morbidity as well as the number of patients seeking treatment. The inclusion criteria included all women, those seeking treatment for post-abortion complications and those with a gestational age of less than 24 weeks. The study team trained those who managed such patients and those who would be available to do this task for the one month period. In addition, in the high volume facilities, more than one PAC provider was trained to collect data. Data collected for each case included patients’ demographics, morbid symptoms, diagnosis on admission, types of treatment received and outcome of the treatment. Data collectors for the PMS were PAC providers at each facility. The PMS was filled out as part of their patient management procedures and many of the questions were filled from provider observations. No patient identifiers were collected. Stringent data quality measures were put in place: supervisors did quality assurance checks to ensure correct data collection protocols were followed during the 30 day data collection period. Fieldwork was conducted between April and May 2012 for both the HFS and PMS.

### Health Professionals Survey (HPS)

Respondents were selected as HPS respondents because of their extensive knowledge about the provision of abortion and post-abortion care in the country. They included researchers, nurses, midwives, clinical officers, lawyers, and obstetrician/gynecologists from different regions of Kenya. A total of 124 key informants were interviewed in-person using a structured questionnaire which covered respondents’ perceptions regarding the type of providers women seek abortions from, the likelihood women will experience complications that require treatment in a facility according to the type of abortion provider used, and the likelihood that women who need treatment will receive it at a health facility. These questions were asked for four major sub-groups within the population: rural poor, rural non-poor, urban poor and urban non-poor. This information was used in the calculation of the multiplier (see section on “Calculating the multiplier”). The HPS interviewers, senior professionals in the field, were trained on the study’s methodology and the questionnaire. Data collection took place between April and August, 2012.

For all study components, informed verbal consent was sought from all participants.

### Other data sources

Other data sources used to conduct the incidence calculations include the 2008/9 Kenya Demographic and Health Survey (KDHS) which provided information on fertility, contraceptive prevalence, unmet need for contraception, birth wantedness and measures of access to health care [14]. Additionally, the 2009 Kenyan Census was used to estimate the number of women aged 15–49 in 2012 for each region and nationally [22]. Finally, the 2005/06 Kenya Integrated Household Budget Survey was used to estimate the proportion of poor/non-poor in urban/rural settings in Kenya [23].

### Estimating the incidence of induced abortion

To estimate the annual incidence of induced abortions in Kenya, the following inputs were used:

1. Estimated number of women treated for post-abortion complications in a typical year and past year in each selected health facility – data retrospectively collected from the HFS.
2. Number of women obtaining abortion care in a 30 day period in each selected health facility – data prospectively collected from the PMS.
3. Estimated likelihood of experiencing a complication from an abortion severe enough to require health care as well as the likelihood of receiving health care for that complication – data collected in the HPS.

#### Number of women treated for abortion complications

The estimated annual total number of women treated for abortion complications was estimated using monthly postabortion cases provided in each facility using both retrospective (HFS) and prospective (PMS) data. These were averaged to provide a best estimate of post-abortion care provision for each facility. Results were weighted according to type of health facility and region to produce nationally representative estimates of the number of women who obtained treatment for abortion complications in health facilities in Kenya in 2012.

Because of the difficulty in classifying patients according to whether they had an induced or a spontaneous abortion, we used an indirect estimation technique to subtract out all spontaneous abortions. Available clinical studies [24, 25] indicate that of all recognized pregnancies, 85 % will end in live births and 15 % in miscarriages. An assumption of AICM is that women with first trimester spontaneous abortions will usually not need medical care in health facilities while late miscarriages (between 13–21 weeks gestation) are likely to require facility-based care. This percent constitutes 2.9 % of all recognized pregnancies, or 3.41 % of all live births [24, 25]. There were an estimated 1,546,000 live births in Kenya in 2012 (applying age-specific fertility rates from the 2008/09 KDHS [14] to women of reproductive age, estimated by projecting from the 2009 Census [22]). Therefore, the estimated number of late miscarriages in 2012 was 52,703, obtained by multiplying the number of live births by 3.41 %.

However, while these women may need care, they may not seek it or obtain it as access to healthcare facilities is very limited in most parts of Kenya and therefore many women may not be able to reach a facility that provides PAC services [19, 26, 27]. Also, others may choose not to seek care out of fear, cultural beliefs or for many other reasons. In addition, the proportion of births supervised by a skilled birth attendant in Kenya is quite low (43.8 %) [14]. Therefore, we assumed, as a measure of access to health care, that the same proportion of women who delivered their last birth in a health facility would be able to seek care for a second trimester miscarriage. Using the 2008/09 KDHS [14], we estimated that 47 % would seek care for a second trimester miscarriage as well as those who did not deliver in a facility but whose reasons for not delivering in a health facility suggest that they would do so when care is needed for a miscarriage (23 %). The calculated estimate for women accessing care for complications of late miscarriages was 70 % nationally (ranging from 62–92 % among regions). We therefore subtracted 70 % of all women with late-term miscarriages from the total number of women treated for abortion complications in 2012 to obtain the number of women treated for abortion complications.

### Calculating the multiplier

Not all women who have induced abortions experience complications and not all those with complications obtain needed care in health facilities [9]. Some have no complications and some have mild complications that do not require treatment in health facilities. Still others have serious complications requiring treatment but do not receive treatment in health facilities. Data from the HPS [19] were used to estimate the proportion who received treatment in facilities among all women who had induced abortions (specifically using the proportions provided by the key informants on a) the types of providers women go to for induced abortions, b) the likelihood of experiencing a complication according to type of provider and c) the likelihood of getting care for abortion complications; all of these probabilities are estimated for the four sub-groups of women (rural/urban and poor/non-poor)). The inverse of this proportion is a factor that, when multiplied by the number of women treated annually in health facilities for complications from induced abortion, yields an estimate of the total number of women who had induced abortions in a given year. Since these estimates are not precise, we developed a range for the multiplier to produce low, medium and high estimates. Taking the HPS-based multiplier as the medium estimate, the low and the high estimates were generated by adding and subtracting one, respectively, from the medium estimate. A higher multiplier suggests safer abortion care and/or less accessibility to health facilities while a lower multiplier suggests less safe abortions and/or more accessibility to the health facilities. The product of the low and high multipliers and the number of women treated annually in health facilities for unsafe abortion complications provides a range around the total number of women having abortions in Kenya in 2012, based on the medium or preferred multiplier. One multiplier estimate was used for Nairobi and the Central region combined because of their closeness in proximity and access to health care. The other multiplier was calculated for the rest of the country as the rest of the country is considered more comparable in terms of access to health care and abortion safety.

### Estimating unintended pregnancy

To calculate the number of unintended pregnancies in 2012 and the unintended pregnancy rates, we first calculated the number of unplanned births. Unplanned births were calculated by applying the proportion of births that were mistimed or unwanted at the time of conception from the 2008/09 KDHS to the estimated number of live births in 2012. The estimates for unplanned births in Kenya were calculated for each region separately from the KDHS. A model-based approach generated from clinical studies of pregnancy loss by gestational age was used to estimate the number of pregnancies ending in miscarriages (including stillbirths) [24, 25]. Based on these studies, spontaneous pregnancy losses are estimated to be approximately 20 % of live births and 10 % of induced abortions. We applied 20 % to the numbers of births and 10 % to the number of abortions to estimate the number of miscarriages occurring in Kenya in 2012.

Combining the numbers of unintended births, unintended pregnancies ending in miscarriages and induced abortions yields an estimate of the overall total number of unintended pregnancies in 2012. Unintended pregnancy rates were then calculated and expressed per 1,000 women of reproductive age (15–49). The number of intended pregnancies was estimated as the total of planned births plus intended pregnancies that ended as miscarriages. Finally, the proportion of pregnancies that were unintended as a percentage of all pregnancies was calculated.

## Results

### Complications from unsafe abortion

We estimated that in 2012 about 157,000 women received care in Kenyan health facilities for complications of induced and spontaneous abortions (Table 2). An estimated 52,700 late miscarriages occurred of which 37,850 received care in health facilities. Thus, about 120,000 women (157,353 minus 37,850) were treated for unsafe abortion complications in health facilities in Kenya in 2012. This means that 12 per 1000 women aged 15–49 received health care for unsafe abortion complications in Kenya in 2012 (Table 3). The incidence of treated induced abortion morbidity ranges from 5 per 1000 women aged 15–49 in the Eastern region to 16 per 1000 women aged 15–49 in the combined Nyanza & Western and the Rift Valley regions.

**Table 2:** Calculating the number of women treated for complications of unsafe abortions in a health facility in 2012, by region (based on women 15–49)

Region	Women treated for abortion complication	Live births among women 15-49^b^	Women with LATE Miscarriages^a^	Women with LATE miscarriages treated in a health facility^c^	Women treated for unsafe abortion complications
Total	157,353	1,545,546	52,703	37,850	119,502
Central & Nairobi	31,506	351,943	12,001	11,005	20,501
Coast & N. Eastern	21,935	209,017	7,127	5,324	16,610
Eastern	12,124	222,435	7,585	5,112	7,011
Nyanza & Western	44,997	375,033	12,789	8,185	36,812
Rift valley	46,791	387,118	13,201	8,224	38,567

^a^Late miscarriages are defined as any spontaneous abortion occurring after 12 weeks and before 24 weeks of pregnancy. Late miscarriages account for 3.41 % of all live births, so these were obtained from multiplying the number of live births by 3.41 %. The number of women with late miscarriages treated at health facility was obtained by multiplying the number of women with late miscarriages as above by the percentage of women who would be treated at a health facility (see description of methodology in text)

^b^Source: Live births were computed by applying the general fertility rate from the 2009 KDHS to the projected (adjusted) number of women of reproductive age from the 2012 population projections from 2009 census

^c^The multiplying factor for the percentage of women with late miscarriages who were treated at heath facilities was estimated from KDHS’s estimated rate of accessing care for 2nd trimester miscarriages

**Table 3:** Women Treated for Abortion Complications in Kenya in 2012 by region

Regions	N&C	C&NE	E	N&W	RV	Total
Annual Abortion Complications						
No. of complications treated						
All abortions	31,506	21,935	12,124	44,997	46,791	157,353
Induced abortions	20,501	16,610	7,011	36,812	38,567	119,502
Rate of complications treated per 1,000 women 15–49						
All abortions	14	17	9	19	19	16
Induced abortions	9	13	5	16	16	12

Note: *N and C* = Nairobi and Central, *C and NE* = Coast and North Eastern, *E* = Eastern, *N and W* = Nyanza and Western, *RV* = Rift Valley

### Incidence of induced abortion

Table 4 shows the estimated number of induced abortions nationally and for the regions. The medium estimate based on the national multiplier indicates that an estimated 464,000 induced abortions occurred in Kenya in 2012. This translates to a national rate of 48 induced abortions per 1,000 women aged 15–49 in 2012 (Table 5). The incidence of induced abortion varied substantially across regions. The abortion rate was highest in the Rift Valley and the combined Nyanza & Western region and lowest in the Eastern region.

**Table 4:** Number of women treated in a health facility for complications of unsafe abortion; and estimated number of induced abortions, by multiplier to account for women not treated for complications; according to region (based on women 15–49)

	No. of women treated	Estimated no. of induced abortions^a^		
Region		Low (multiplier = 2.88)	Medium (multiplier = 3.88)	High (multiplier = 4.88)
Total^b^	119,502	344,430	463,932	583,434
Central & Nairobi	20,501	49,408	69,909	90,411
Coast & N. Eastern	16,610	49,499	66,109	82,719
Eastern	7,011	20,894	27,905	34,917
Nyanza & Western	36,812	109,701	146,513	183,326
Rift valley	38,567	114,929	153,495	192,062

^a^We used two regional multipliers; one for Nairobi and Central region combined (Low = 2.41; Medium =3.41 and High = 4.41) and another multiplier for the rest of the country’s regions (Low = 2.98; Medium =3.98 and High = 4.98)

^b^Generated as a sum of the regional estimates

**Table 5:** Abortion rate and ratio for each region and for Kenya in 2012, by level of multiplier (based on women 15–49)

Region	Women of Reproductive Age	Abortion Rate			Abortion Ratio		
		Low	Medium	High	Low	Medium	High
Total	9,599,666	36	48	61	22	30	38
Central & Nairobi	2,185,980	23	32	41	14	20	26
Coast & N. Eastern	1,298,244	38	51	64	24	32	40
Eastern	1,381,582	15	20	25	9	13	16
Nyanza & Western	2,329,398	47	63	79	29	39	49
Rift valley	2,404,461	48	64	80	30	40	50

Source: Kenya National Bureau of Statistics, National Population and Housing Census, 2009. These numbers were projected to 2012 using the 1999–2009 population growth rate. Abortion rate is the number of induced abortions per 1,000 women aged 15–49 per year. Abortion ratio is the number of induced abortions per 100 live births

The national abortion ratio of 30 per 100 live births implies that about one in three pregnancies ended in an induced abortion in 2012. The Rift Valley and the combined Nyanza and Western region had abortion ratios of 39 and 40 per 100 live births, respectively. The lowest abortion ratio, 13 per 100 live births, was found in the Eastern region.

### Induced abortion incidence by age groups

**Fig. 1:**
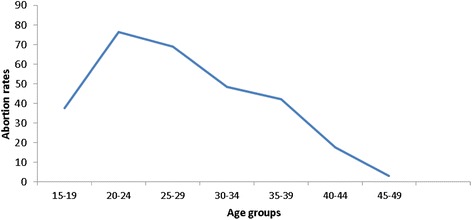
Induced Abortion Rates per 1,000 women aged 15–49, Kenya, 2012

The age-specific abortion rates exhibited an inverted J-shaped pattern (Fig. 1). The abortion rate was highest among women aged 20–24 and lowest among women aged 45–49. The abortion rate rose from 38 per 1000 women among adolescents (15–19 year-olds) to 76 per 1000 among women aged 20–24, before declining slightly to 69 among 25–29 year-olds. Large declines were seen above age 30–34 with the lowest rate among women aged 45–49 (a rate of 3).

### Incidence of unintended pregnancies

Of all pregnancies, about half (49 %) were unintended (Table 6). The unintended pregnancy rate was 120 per 1,000 women 15–49 in 2012. Forty-one percent of unintended pregnancies ended as abortions. The combined regions of Coast and North Eastern had the highest proportion of unintended pregnancies ending as abortion, followed by Rift Valley and the Nyanza & Western, while that proportion was lowest in the Eastern region.

**Table 6:** Number and rate of pregnancies, unintended pregnancy rate, percent of pregnancies unintended and percent of unintended pregnancies ending as abortions by region, Kenya, 2012

	Total pregnancies^a^	Pregnancy Rate^b^	Unintended Pregnancy Rate^c^	% of pregs that were unintended	% of unintended pregs ending as abortions
Total	2,366,665	264	120	48.6	40.5
Central & Nairobi	499,886	245	93	40.8	34.6
Coast & N. Eastern	323,712	265	84	33.7	60.8
Eastern	297,817	234	97	44.9	21.0
Nyanza & Western	611,334	283	145	55.2	43.5
Rift valley	633,916	282	142	54.0	45.0

^a^Total pregnancies include births, induced abortions, and miscarriages

^b^Number of pregnancies per year per 1,000 women aged 15–49

^c^Number of unintended pregnancies per year per 1,000 women aged 15–49

Source: Population estimates for 2012 are based projections of 2009 census. Age specific fertility rates (ASFRs) obtained from the 2008/9 Kenya Demographic and Health Survey (KDHS) were applied to the projected 2012 population of women by five-year age groups to estimate number of births in 2012. Proportion of unplanned births (unwanted or mistimed, also from the 2008/9 KDHS) was applied to total 2012 projected number of births (nationally and by region) to obtain the number of unplanned births in 2012

## Discussion

This study provides the first nationally-representative estimates of the incidence of induced abortion in Kenya. The last survey which was conducted in public facilities only estimated the abortion rate to be 45 per 1000 women of reproductive age. In the current study, an estimated 464,000 women had induced abortions in Kenya in 2012 and this translated to an abortion rate of 48 per 1000 women of reproductive age. While Kenya’s abortion rate is similar to Uganda’s 54 per 1000 women of reproductive age, it is much higher than the rate in Ethiopia (23), Malawi (23), Rwanda (25), Burkina Faso (25) [6, 7, 20, 28] and the whole of the Eastern Africa region (38) [1]. Kenya’s abortion rate is consistent with the high level of unmet need for family planning (26 %) in the country [14]. The results also suggest that the new abortion law in Kenya has not translated into improved service delivery.

It is almost five years since Kenya approved a new constitution that affirmed women’s rights to reproductive health by easing the earlier abortion restrictions [16]. However, two years later, women were still showing up at health facilities seeking PAC in severe conditions following unsafe termination of pregnancy [29]. Despite increased legal abortion provision in some areas, the provisions in the constitutions have not been operationalized and this leaves both the women and health providers unsure whether they are protected by the new constitution if they sought or provided abortion services respectively. In a qualitative community based study done in 2012 in 3 counties in Kenya, it was obvious that many women were not clear on whether they were any legal exceptions on abortions under certain circumstances [17].

Substantial variation exists with regard to abortion incidence across the country. The Rift Valley region, the combined Nyanza & Western region, and the combined Coast & North Eastern regions have the highest abortion rates. These high rates are consistent with the high levels of unwanted fertility in these regions as estimates show in the KDHS 2008/09 report [14]. Variations are somewhat expected because of differences in cultural norms, education levels, socio-economic factors, sexual and contraceptive behavior and fertility preferences, among other factors. For instance, the low abortion rate in the Eastern region can be partly explained by its above-average contraceptive prevalence (52 % for any method and 44 % for modern methods) among married women compared to 37–47 % in the other two regions [14]. The Eastern region also has the highest median duration of exclusive breastfeeding compared to the rest of the country which prolongs lactational amenorrhea and thereby delays conception, reducing the time of exposure to pregnancy [14]. An additional possible reason for the region’s low abortion incidence is that one of the biggest towns in the Eastern region, Machakos, is near to Nairobi and it is possible that women in Machakos seek post abortion services in Nairobi. This would result in underestimation of the abortion rate in Eastern region, and overestimation of the rate in Nairobi. More research is required to understand why variations exist in the regions.

Age-specific data on abortion in Kenya showed that rates were high among adolescents, peaked among women aged 20–24, slightly decreased among the women aged 25–29, before decreasing steadily among older women. The high rate of abortion among women aged 15–24 is consistent with the high level of unmet need for family planning among this age-group. For instance, 30 % of married adolescents and married women 20–24 had an unmet need for contraception. Unmet need was 21 % among unmarried sexually active 15–24 year-olds [14].

Of all pregnancies in 2012, half were unintended. This percentage is twice what is found in a study in Nairobi conducted between 2009/10 which based the estimates on pregnancy intention [30]. It is not surprising that our estimates are higher because our calculation also takes into account abortions. Furthermore, there may be reporting bias in the methods used by Ikamari et al. as women are more likely to report having wanted the child if they carried the child to term and women in their sample were asked to report on intendedness only of pregnancies they carried to term. Results from the current study suggest unsafe abortion uses substantial health care resources as an estimated 120,000 women age 15–49 are being treated for complications of induced abortion each year. It appears that national policy priorities are not in line with women’s health needs. A recent report also highlights the decreased budget allocation to health and the declining attention to financing reproductive health in Kenya [31]. The budget allocation towards health in Kenya in 2013/2014 was 34.7 billion Ksh (~ USD 386 million) compared to 55.1 billion Ksh (~ USD 613.5 million) in 2012/2013 and this is far below the Abuja declaration of 15 % of the total domestic budget [32].

The current study has its strengths and limitations. Its key strength is the use of a well-recognized methodology (the Abortion Incidence Complications Methodology (AICM) and the Prospective Morbidity Survey (PMS)) [19] which gives estimates based on a nationally representative sample that included both public and private health facilities. One limitation is the data used by the indirect methodology in estimating the number of late spontaneous abortions. The incidence of late spontaneous abortions is estimated based on biological patterns of pregnancy loss documented by clinical trials done before in early 1980’s in a developed country. It is possible that the incidence of spontaneous abortion may be different today in a developing country context due to factors such as HIV/AIDS, malaria, malnutrition and violence. However, there is no alternative data source on spontaneous pregnancy loss by gestation. Another limitation is the assumption that women with first trimester spontaneous abortions will not seek care, while all women with late miscarriages will want to seek care but only successfully do so at a rate equal to women delivering in hospitals, as well as those not delivering in hospitals. These assumptions may not accurately capture the true incidence of care-seeking among women having miscarriages in either the first or second trimester which vary by access, ethnicity or geography. The variables used to generate the multiplier were based on opinions of experts. Lastly, while these are informed opinions of a large group of knowledgeable key informants, and the only available approach for estimating these factors, they can provide only approximate measures. Because of these limitations, the findings should be regarded as estimates rather than exact measures.

## Conclusion

The incidence of induced abortion in Kenya and the proportion of women seeking care for unsafe abortion complications show that women are experiencing unwanted pregnancies and health complications from ending those pregnancies in unsafe ways. These results support the need for increased access to effective modern contraceptive services among women in Kenya in order to reduce unintended pregnancies. Interventions to improve family planning services and increase contraceptive use are therefore needed. The higher abortion rate among young women suggests a need for programs that specifically address these women’s needs for information and services relating to pregnancy prevention. This study calls for further research to understand what potential policies and interventions can be put in place to help women achieve their reproductive goals without endangering their health. Finally, there should be concerted effort on the part of the government to ensure adequate post abortion care services and improved access to safe abortion to replace unsafe clandestine procedures within the limit of the law.

## Endnote

^1^All level III-VI are expected by the MOH to offer PAC services. In this study we also included level II facilities that provided maternity services as they would have the potential to offer PAC services.

## Abbreviations

AICM: Abortion Incidence Complications Methodology
HFS: Health Facility Survey
HMIS: Health Management Information System
HPS: Health Professionals Survey
KDHS: Kenya Demographic and Health Survey
KEMRI: Kenya Medical Research Institute
KEPH: Kenya Essential Package for Health
MOH: Ministry of Health
PAC: Post-abortion Care
PMS: Prospective Morbidity Survey
SSA: Sub-Saharan Africa
WHO: World Health Organization
